# Malarone® induced pancreatitis and alopecia in a dog: a case report

**DOI:** 10.1186/s12917-019-2056-9

**Published:** 2019-09-02

**Authors:** Hyeong-Il Choi, Hui-Yeon Ko, In-Sik Shin, Ha-Jung Kim

**Affiliations:** 1 Department of Internal Medicine, College of Veterinary Medicine, Chonnam National University, Yongbong-ro, Buk-gu, Gwangju, 61168 Korea; 2 BK21 project team, College of Veterinary Medicine, Chonnam National University, Gwangju, 61168 Korea

**Keywords:** Adverse drug reaction, Alopecia, Malarone®, Pancreatitis

## Abstract

**Background:**

Malarone**®** is a drug used for the treatment of malaria in humans. This drug is also particularly effective in the treatment of canine *Babesia gibsoni* infections. Malarone**®** is rarely used in dogs, and its adverse effects have not been widely reported. Its mechanism of action is related to the inhibition of cytochrome b and electron transport in the cell. This is the first known report of the development of acute pancreatitis and alopecia in a dog following the administration of Malarone**®**.

**Case presentation:**

A 3-year-old, intact, female Maltese was referred to our clinic with intermittent vomiting and sudden, generalized alopecia. Two months previously, the dog had been prescribed Malarone**®** for the treatment of a suspected *B. gibsoni* infection. The dog was evaluated using hematology, radiography, ultrasonography, a PCR for *Babesia* detection, and a canine pancreatic lipase immunoreactivity (cPLI) assay. The result of the PCR test was negative, whereas the cPLI assay yielded a positive result. Dermatologic examination revealed bacterial infection with hair cycle arrest.

**Conclusions:**

Based on these findings, drug-induced acute pancreatitis and alopecia with superficial pyoderma were diagnosed. Malarone**®** may induce severe adverse reactions in dogs. Therefore, careful monitoring for adverse effects is required when using Malarone**®** in dogs.

## Background

Malarone**®** (GlaxoSmithKline, London, UK), which is commercially available for the treatment of malaria in humans, contains atovaquone (ATV) and proguanil (PG). Atovaquone is well tolerated and has a broad-spectrum of antiprotozoal activity in dogs [1]. In particular, this drug is effective in the treatment of canine *Babesia gibsoni* infections. Proguanil is a highly protein-bound molecule that has been used in combination with ATV for the treatment of *B. gibsoni* infections [2, 3].

The most common adverse effects in people receiving ATV for the treatment of malaria are abdominal pain, maculopapular rash, nausea, diarrhea, vomiting, and headache; approximately 10–35% of patients experience at least one of these symptoms [2, 4]. Furthermore, several cases of reversible alopecia have been reported in humans receiving high doses of PG [5].

Malarone**®** is rarely used in dogs, and its adverse effects have not been widely reported. One study reported severe gastrointestinal effects secondary to the use of Malarone**®** in dogs [1]. To the best of our knowledge, this case report is the first to describe the development of acute pancreatitis and alopecia in a dog following the administration of Malarone**®**.

## Case presentation

A 3-year-old, intact, female Maltese was referred to our clinic with vomiting, anorexia, and sudden alopecia. Two months previously, the dog had been prescribed Malarone**®** (atovaquone, 13.3 mg/kg PO BID; GlaxoSmithKline, London, UK) elsewhere for the treatment of a suspected *B. gibsoni* infection. She had been administered the drug up until the time of presentation at our clinic.

**Fig. 1:**
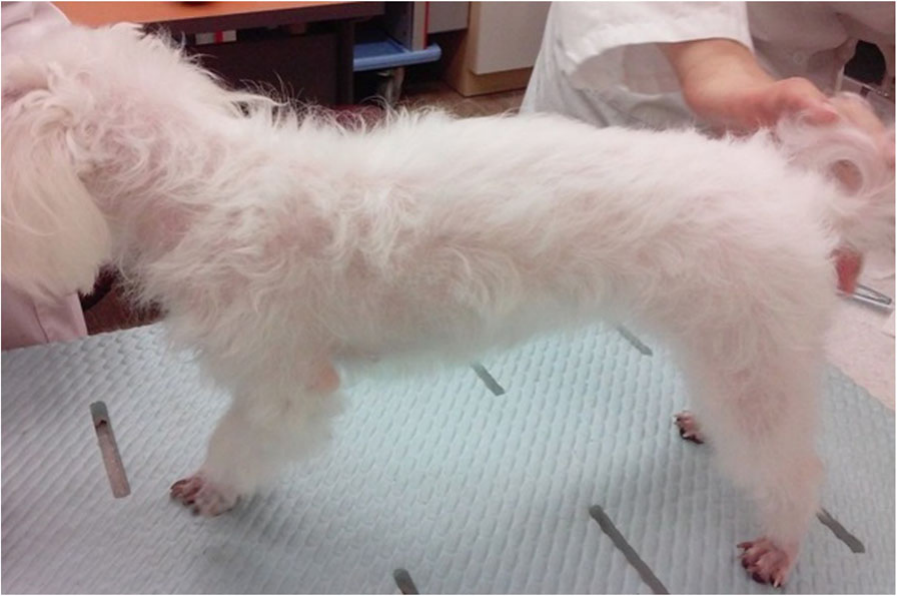
Photographs of the dog. Hair loss is shown occurring mainly around the dorsum and neck

On physical examination, mild depression, dehydration, and generalized alopecia were evident (Fig. 1).

No Babesia organisms were seen on blood smear examination. The results of a real-time PCR test for *Babesia spp.* (IDEXX Reference Laboratories, Westbrook, ME, USA) were also negative. Serum biochemistry analysis indicated mildly increased concentrations of amylase (2500 U/*l*; reference range 500–1500 U/*l*) and lipase (4420 U/*l*; reference range 200–1800 U/*l*). Thoracic and abdominal radiographs, as well as an abdominal ultrasound, showed no remarkable findings. A canine pancreatic lipase immunoreactivity assay (SNAP cPLI kit; IDEXX Reference Laboratories, Westbrook, ME, USA) yielded abnormal results, consistent with pancreatitis. Based on the clinical symptoms, results of the SNAP cPLI test, and abnormalities noted on the serum biochemistry analysis, a diagnosis of acute pancreatitis was made.

Cytology performed on skin scrapings and fungal cultures of the samples were negative. Using acetate tape, samples were collected for cytology from each of the affected areas. Numerous cocci were identified on the samples obtained from the alopecic flank. Therefore, the dog was diagnosed with alopecia with secondary superficial pyoderma. Both the pancreatitis and the alopecia were suspected to be “adverse drug reactions” (ADRs). Using a previously created scoring system, we calculated the likelihood of these conditions being the result of an ADR [6]. Based on the history and the results of a physical examination, this dog was assigned an ADR probability score of + 5 (Table 1), which suggests that the clinical signs could have been caused by the Malarone**®**.

**Table 1:** Adverse drug reactions (ADRs) probability scale [6]

Questions	Yes	No	Don’t know	Score ^a)^
1. Are there previous conclusive reports on this reaction?	+ 1	0	0	0
2. Did the adverse event appear after the suspected drug was administered?	+ 2	−1	0	+ 2
3. Did the adverse reaction improve when the drug was discontinued or a specific antagonist was administered?	+ 1	0	0	0
4. Did the adverse reaction reappear when the drug was readministered?	+ 2	−1	0	0
5. Are there alternative causes (other than the drug) that could on their own have caused the reaction?	−1	+ 2	0	+ 2
6. Did the reaction reappear when a placebo was given?	−1	+ 1	0	0
7. Was the drug detected in the blood (or other fluids) in concentrations known to be toxic?	+ 1	0	0	0
8. Was the reaction more severe when the dose was increased, or less severe when the dose was decreased?	+ 1	0	0	0
9. Did the patient have a similar reaction to the same or similar drug in any previous exposure?	+ 1	0	0	0
10. Was the adverse event confirmed by any objective evidence?	+ 1	0	0	+ 1
Total score ^b)^				+ 5

a) Score was applied to the dog in this case

b) Total score is the sum of all subcategory scores. The relationship is categorized as ‘Definite’ if the score is greater than 8, ‘Probable’ if the score is 5 to 8, ‘Possible’ if the score is 1 to 4, and ‘Doubtful’ if the score is 0

Initial treatment consisted of intravenous fluid therapy (0.9% NaCl), fresh frozen plasma (Special B canine plasma; Korea Animal Blood Bank), cefixime hydrate (10 mg/kg PO BID; Cefixime, Withus Pharm, Anseong, South Korea), and enrofloxacin (5 mg/kg PO BID; Baytril Flavour Tablets, Bayer, KVP Pharm, Kiel, Germany) to address pancreatitis, as well as melatonin (2 mg PO BID; Circadin, Neurim Pharm, Zug, Switzerland) to treat alopecia. After 10 days, the dog’s clinical signs improved and the hair began to regrow slowly. Serum lipase (558 U/*l*; reference range 200–1800 U/*l*) and amylase (636 U/*l*; reference range 500–1500 U/*l*) activities measured within normal limits.

## Discussion and conclusions

This study describes a clinical case of alopecia and pancreatitis in a dog believed to be secondary to treatment with the antiprotozoal drug, Malarone**®**. The dog had been presumptively diagnosed with Babesiosis and was on a prolonged course (approximately 2 months duration) of low-dose Malarone**®** before presenting with vomiting and sudden hair loss. There is minimal information in the literature concerning this drug and the adverse effects associated with its use.

The prevention of human malaria using higher doses of ATV and PG has been associated with adverse gastrointestinal effects and increases in liver transaminase and bilirubin in approximately 15% of patients in human [2]. In dogs, only a few reports have described the side effects of Malarone**®**. For instance, it has been reported that a 2.5:1 combination of ATV and PG results in diarrhea and vomiting in dogs [1]. The standard dose of ATV reported to be17–25 mg/kg twice daily for 10 days in dogs. In spite of the lower dose (13.3 mg/kg twice daily), the dog showed side effects with long term therapy. The dog had been dosed for 2 months, which is approximately six times longer than the normal course of therapy.

Pharmacology-related adverse effects result from excessive responses to a drug; these effects are dose-dependent, predictable, and are caused by a known pharmacological property of the agent. In addition, there may be an immunological basis to such effects [7]. In this case, the dog’s symptoms appeared to be related to dose-dependent pharmacological side effects. The mechanism of action of ATV against protozoa is believed to involve the inhibition of cytochrome b and electron transport in the cell. When used with ATV, PG enhances its actions and helps to collapse the mitochondrial membranes of the protozoa [1].

In humans, attempts have been made to systematize the assessment of causality of ADRs, applying operation definitions such as those proposed by the Naranjo Score (Table 1) [6]. According to this scoring system, the development of pancreatitis or gastrointestinal (GI) signs and alopecia belong in the category of a “probable” ADR.

In the field of human medicine, the use of ATV has not been clearly associated with the development of pancreatitis and alopecia. Instead, GI signs including vomiting and diarrhea and dermatologic problems including rash and pruritus have been reported [8]. Based on the literature [1, 8], and on the dramatic clinical signs that developed during treatment with Malarone**®** in this case, we presumed an association between the two entities. Additional clinical studies investigating the use of ATV in veterinary patients are required to assess its efficacy and its associated side effects.

In conclusion, Malarone**®** may induce severe adverse reactions in dogs, including alopecia and acute pancreatitis. Therefore, a careful monitoring for adverse effects is required during the administration of Malarone**®** in dogs. To our knowledge, this is the first report of pancreatitis and alopecia developing as severe side effects to the usage of Malarone**®** in a dog.

## Data Availability

All data supporting our findings are included in this study.

## Abbreviations

ATV: Atovaquone
*B. gibsoni*: *Babesia gibsoni*
bid: Bis in die
cPLI: Canine pancreatic lipase immunoreactivity
PCR: Polymerase chain reaction
PG: Proguanil
PO: Per os
